# Effect of Temperature and Selection for Growth on Intracellular Lipid Accumulation and Adipogenic Gene Expression in Turkey Pectoralis Major Muscle Satellite Cells

**DOI:** 10.3389/fphys.2021.667814

**Published:** 2021-06-01

**Authors:** Jiahui Xu, Gale M. Strasburg, Kent M. Reed, Sandra G. Velleman

**Affiliations:** ^1^Department of Animal Sciences, The Ohio State University, Wooster, OH, United States; ^2^Department of Food Science and Human Nutrition, Michigan State University, East Lansing, MI, United States; ^3^Department of Veterinary and Biomedical Sciences, University of Minnesota, St. Paul, MN, United States

**Keywords:** adipogenic potential, selection for growth, muscle, satellite cell, temperature

## Abstract

As multipotential stem cells, satellite cells (SCs) have the potential to express adipogenic genes resulting in lipid synthesis with thermal stress. The present study determined the effect of temperature on intracellular lipid synthesis and adipogenic gene expression in SCs isolated from the pectoralis major (p. major) muscle of 7-day-old fast-growing modern commercial (NC) turkeys compared to SCs from unselected slower-growing turkeys [Randombred Control Line 2 (RBC2)]. Since proliferating and differentiating SCs have different responses to thermal stress, three incubation strategies were used: (1) SCs proliferated at the control temperature of 38°C and differentiated at 43° or 33°C; (2) SCs proliferated at 43° or 33°C and differentiated at 38°C; or (3) SCs both proliferated and differentiated at 43°, 38°, or 33°C. During proliferation, lipid accumulation increased at 43°C and decreased at 33°C with the NC line showing greater variation than the RBC2 line. During proliferation at 43°C, peroxisome proliferator-activated receptor-γ (*PPAR*γ) and neuropeptide-Y (*NPY*) expression was reduced to a greater extent in the NC line than the RBC2 line. At 33°C, expression of *PPAR*γ, *NPY*, and CCAAT/enhancer-binding protein-β (*C/EBP*β) was upregulated, but only in the RBC2 line. During differentiation, both lines showed greater changes in lipid accumulation and in *C/EBP*β and *NPY* expression if the thermal challenge was initiated during proliferation. These data suggest that adipogenic gene expression is more responsive to thermal challenge in proliferating SCs than in differentiating SCs, and that growth-selection has increased temperature sensitivity of SCs, which may significantly affect breast muscle structure and composition.

## Introduction

Satellite cells (SCs) are a heterogenous stem cell population (Schultz, 1974; Kuang et al., 2007; Biressi and Rando, 2010; Smith et al., 2013; Tierney and Sacco, 2016), functioning in hypertrophic growth of post-hatch muscle (Moss and Leblond, 1971; Cardiasis and Cooper, 1975). As multipotential stem cells (Asakura et al., 2001; Shefer et al., 2004), SCs with appropriate external stimuli have the potential to spontaneously convert to other lineages such as adipocytes (Asakura et al., 2001; Csete et al., 2001; Shefer et al., 2004; Redshaw and Loughna, 2012). Environmental temperature is an external stimulus that has been shown to alter the cellular function and fate of SCs in chickens (Halevy et al., 2001; Harding et al., 2015; Piestun et al., 2017; Patael et al., 2019) and turkeys (Clark et al., 2016, 2017; Xu et al., 2021). Piestun et al. (2017) and Patael et al. (2019) showed when chickens were challenged with chronic heat stress immediately after hatch for 2 weeks, proliferation of SCs was suppressed while fat deposition was increased in the pectoralis major (p. major; breast) muscle. In contrast, continuous cold stress for the same period of time increased chicken p. major muscle satellite cell (pmSC) proliferation without affecting fat content of the breast muscle (Patael et al., 2019). *In vitro* studies with cultured pmSCs from both chickens (Harding et al., 2015) and turkeys (Clark et al., 2017) have demonstrated an increased amount of intracellular lipid with heat stress. The increased lipid content was hypothesized to arise from the conversion of pmSCs to an adipogenic-like lineage under the thermal stress.

Conversion of SCs to an adipogenic-like lineage is regulated by adipogenic regulatory factors like peroxisome proliferator-activated receptor-gamma (*PPAR*γ) (Tontonoz et al., 1994; Rosen et al., 1999; Vettor et al., 2009) and some members in the CCAAT/enhancer-binding protein (C/EBP) family (Freytag et al., 1994; Wu et al., 1995; Rosen and Macdougald, 2006) as well as the kruppel like factor (KLF) family (Rosen and Macdougald, 2006; Tahmasebi et al., 2013; Wu and Wang, 2013). The protein *C/EBP*β is expressed during the initiation stages of adipogenesis leading to an adipogenic differentiation pathway (Ntambi and Young-Cheul, 2000). The expression of *C/EBP*β promotes the transcription of *PPAR*γ during intermediate stages of adipogenic differentiation (Wu et al., 1995; Clarke et al., 1997; Ntambi and Young-Cheul, 2000). The *PPAR*γ is required to stimulate adipogenesis of multiple types of cells including myoblasts (Hu et al., 1995; Holst et al., 2003) and pre-adipocytes (Chawla et al., 1994; Bastie et al., 1999). After being challenged with chronic heat stress immediately after hatch, chicks exhibit increased intramuscular fat deposition coupled with increased *C/EBP*β expression in the breast muscle (Piestun et al., 2017; Patael et al., 2019). Furthermore, both heat and cold stress modulates the adipogenic characteristics of pmSCs in chickens (Harding et al., 2015) and turkeys (Clark et al., 2017) through altering the expression of *C/EBP*β and *PPAR*γ. Taken together, these findings suggest post-hatch thermal stress may affect lipid homeostasis of pmSCs, in part, by modifying the expression of adipogenic transcriptional factors. The family of KLFs are zinc-finger transcriptional factors (Kaczynski et al., 2003) consists of members involved in adipogenesis (Wu and Wang, 2013). Some members like KLF1 (Zhang et al., 2015) and KLF7 (Kawamura et al., 2006; Cho et al., 2007) are mainly expressed in pre-adipocytes, and inhibit adipogenic differentiation. Thus, KLF1 and KLF7 are regarded as pre-adipocyte markers.

The secretory peptide, Neuropeptide-Y (NPY), functions as an extracellular signal regulating adipogenesis by interacting with NPY receptors (Yang et al., 2008; Baker et al., 2009; Shipp et al., 2016). The expression of *NPY* promotes lipid deposition by promoting adipogenesis (Kuo et al., 2007; Baker et al., 2009; Zhang et al., 2015; Shipp et al., 2016) and inhibiting lipolysis (Valet et al., 1990; Park et al., 2014; Zhang et al., 2014; Liu et al., 2017). At the transcriptional level, *NPY* was the gene in turkey pmSCs most downregulated by cold stress during differentiation (Reed et al., 2017). With heat stress, increased expression of *NPY* and its receptors was observed in turkey pmSCs (Clark et al., 2018). Because NPY promotes chicken adipocyte adipogenesis through regulating *PPAR*γ and *C/EBP*α expression (Zhang et al., 2015), it may act as an upstream signal regulating adipogenic transcriptional factors during thermal-stress-induced adipogenic response in avian SCs.

Birds are homotherms and maintain their body temperature in a limited range (Yahav, 2000, 2015). For newly hatched poults, it is difficult to control body temperature due to an immature thermal regulatory system (Dunnington and Siegel, 1984; Modrey and Nichelmann, 1992; Shinder et al., 2007). Furthermore, SCs exhibit their peak mitotic activity (Mozdziak et al., 1994; Halevy et al., 2000) and temperature sensitivity (Halevy et al., 2001; Piestun et al., 2017; Patael et al., 2019; Halevy, 2020) during the first week after hatch. Newly hatched birds are frequently exposed to both hot and cold temperatures during handling and transportation from hatcheries to growing facilities. Heat stress during this period increases fat deposition in chicken breast muscle (Zhang et al., 2012; Patael et al., 2019; Halevy, 2020), which parallels the increased intracellular lipid content of cultured pmSCs in heat challenged chickens (Harding et al., 2015) and turkeys (Clark et al., 2017). Thus, early age thermal stress may detrimentally affect breast muscle structure and composition by altering the adipogenic properties of pmSCs.

Faster-growing chickens produce more metabolic heat than shower-growing lines because of a higher metabolic rate (Konarzewski et al., 2000). In addition, faster-growing heavy weight meat-type chickens have reduced capacity for heat dissipation due to their reduced breast muscle capillary density (Hoving-Bolink et al., 2000; Yahav, 2000; Havenstein et al., 2003; Joiner et al., 2014). Thus, when exposed to thermal stress, faster-growing poultry have reduced thermal tolerance (Berrong and Washburn, 1998; Yahav, 2000; Chiang et al., 2008), resulting in prolonged negative effects on breast muscle structure and growth (Joiner et al., 2014; Piestun et al., 2017; Patael et al., 2019). The proliferation rate and differentiation level of pmSCs in growth-selected faster-growing turkeys is more responsive to both heat and cold stress than non-selected slower-growing turkeys (Clark et al., 2016; Xu et al., 2021). In addition, proliferating pmSCs are more sensitive to both heat and cold stress than differentiating pmSCs (Xu et al., 2021).

The objective of the current study was to determine the effects of both heat and cold stress and selection for growth on intracellular lipid accumulation and adipogenic regulatory gene expression in pmSCs from 7-day-old Nicholas Commercial (NC) turkeys compared to those from Randombred Control Line 2 (RBC2) turkeys. The commercial turkeys used in this study were selected for growth characteristics including high growth rate and heavy breast muscling, whereas the RBC2 turkeys represent commercial turkeys in the mid-1960s (Nestor et al., 1969) and are slower-growing turkeys without heavy breast muscling. The temperature regimens used in the current study included all the possible combinations of heat and cold stress during pmSC proliferation and/or differentiation as reported by Xu et al. (2021). These regimens allowed a complete assessment of the effect of thermal stress on the adipogenic characteristics of the pmSCs from the two genetic lines. Data from the current study will provide insight on how thermal stress and selection for growth characteristics affects the synthesis of lipid by pmSCs during proliferation and differentiation *in vitro*. These findings have potential application in the development of management strategies to control lipid production by pmSCs which has been reported to affect the fiber organization and cellular properties of the breast muscle (Velleman et al., 2014; Patael et al., 2019).

## Materials and Methods

### Pectoralis Major Muscle Satellite Cell Culture

Satellite cells were isolated from the p. major muscle of 7-day-old RBC2 turkeys and 7-day-old NC turkeys based on the method of Velleman et al. (2000). All the cells were passaged to a fourth pass and stored in liquid nitrogen until use.

Three different experimental strategies were used in the present study: (1) pmSCs proliferated at 38°C (control) and differentiated at 43° or 33°C; (2) pmSCs proliferated at 43° or 33°C and differentiated at 38°C; or (3) pmSCs both proliferated and differentiated at 43°, 38°, or 33°C (Table 1). Cell culture procedures were based on the method of Velleman (2014). In brief, 15,000 of cells were plated in each well of 24-well plates (Greiner Bio-One, Monroe, NC, United States) in Dulbecco’s Modified Eagle’s Medium (DMEM) (Sigma-Aldrich, St. Louis, MO, United States) plating medium containing 10% chicken serum (Gemini Bio-Products, West Sacramento, CA, United States), 5% horse serum (Gemini Bio-Products), 1% antibiotics-antimycotics (Gemini Bio-Products), and 0.1% gentamicin (Gemini Bio-Products). For cell attachment, the cells were initially incubated in a 95% air/5% CO2 incubator (Thermo Fisher Scientific, Waltham, MA, United States) at 38°C for 24 h. After attachment, the plating medium was replaced with McCoy’s 5A growth medium (Sigma-Aldrich) containing 10% chicken serum (Gemini Bio-Products), 5% horse serum (Gemini Bio-Products), 1% antibiotics-antimycotics (Gemini Bio-Products), and 0.1% gentamicin (Gemini Bio-Products). The growth medium was refreshed every 24 h for 72 h. After 72 h of proliferation, the growth medium was replaced with a DMEM differentiation medium containing 3% horse serum, 1% antibiotics-antimycotics, 0.1% gentamicin, 0.1% gelatin, and 1 mg/mL bovine serum albumin (BSA) (Sigma-Aldrich) for 72 h of differentiation. The differentiation medium was changed every 24 h for 72 h.

**TABLE 1:** Incubation strategies during satellite cell proliferation and differentiation.

	Temperature (°C)	
	Proliferation^1^	Differentiation^2^
Regimen 1	38	43
	38	38
	38	33
Regimen 2	43	38
	38	38
	33	38
Regimen 3	43	43
	38	38
	33	33

*^1^From 0 to 72 h of proliferation.*

*^2^From 0 to 72 h of differentiation.*

### Intracellular Lipid Content Measurement

AdipoRed fluorochrome (Lonza, Walkersville, MD, United States) was used to quantify intracellular lipid content in pmSCs every 24 h during 72 h of proliferation and every 24 h during 72 h of differentiation according to the manufacturer’s procedure. One plate from each treatment group was collected and was assayed at each sampling time. First, the medium was removed from each plate. Second, phosphate buffered saline (PBS) (pH 7.08), which contained 137 mM of NaCl, 2.68 mM of KCl,1.47 mM of KH_2_PO_4_, and 7.81 mM of Na_2_HPO_4_, was used to rinse the plate twice. Third, another 1 mL of PBS containing 30 μL of AdipoRed was added to each well. One well with only 1 mL PBS without AdipoRed was used as a negative control. After incubating for 15 min, the AdipoRed levels were quantified based on the fluorescence absorbance at a wavelength of 485 nm in a plate reader (Fluoroskan Ascent FL, ThermoElectron Co., Waltham, MA, United States). The AdipoRed assay was repeated in three independent cultures with four wells per treatment group per cell line.

### Gene Expression Analysis

Effect of thermal stress and selection for growth on the expression of adipogenic regulatory factors was determined. Cells were cultured according to the temperature strategies outlined in Table 1 and was sampled at 72 h of proliferation and at 48 h of differentiation. Extraction of total RNA from each sample was conducted using RNAzol (Molecular Research Center, Cincinnati, OH, United States) based on manufacturer’s procedures. The concentration of each RNA sample was quantified with a spectrophotometer (NanoDrop^TM^ ND-1000, Thermo Fisher Scientific). Reverse transcription was performed using Moloney Murine Leukemia Virus Reverse Transcriptase (M-MLV) (Promega, Madison, WI, United States) to produce cDNA from total RNA. Real-Time Quantitative PCR (RT-qPCR) was conducted using DyNAmo Hot Start SYBR Green qPCR kit (Thermo Fisher Scientific) to quantify the expression of *C/EBP*β, *KLF1*, *KLF7*, *NPY*, and *PPAR*γ. Primers for *KLF1* and *KLF7* were designed using primer-BLAST tool on the website of National Center for Biotechnology Information. The amplification specificity of each primer was validated by sequencing the PCR product. Primers for *C/EBP*β, *NPY*, *PPAR*γ, and glyceraldehyde-3-phosphate dehydrogenase (*GAPDH*) (a normalizer) were previously designed and specificity confirmed (Velleman et al., 2014; Clark et al., 2017, 2018). Information of all the genes and primers is listed in Table 2. The RT-qPCR reaction was the following: 94°C for 15 min; amplification for 35 cycles; and final elongation at 72°C for 5 min in a DNA Engine Opticon 2 real-time machine (Bio-Rad, Hercules, CA, United States). Each amplification cycle was as follows: sample cDNA was denatured for 30 s at 94°C; annealed for 30 s at 60°C for *C/EBP*β and *KLF1* or at 55°C for *GAPDH*, *KLF7*, *NPY*, and *PPAR*γ; and elongated for 30 s at 72°C. According to the method of Liu et al. (2006), a standard curve of each gene was generated using serial dilutions of purified PCR products. Arbitrary concentrations from 1 to 100,000 were assigned to each serial dilution. Since the concentration of each amplified cDNA sample was within the concentration range of the corresponding stander curve, the arbitrary molar concentration of each amplified sample was calculated according to the threshold cycle. The arbitrary molar concentration of each cDNA sample was normalized using *GAPDH*. The RT-qPCR for each gene was repeated in three independent cultures with 12 wells per treatment group per cell line.

**TABLE 2:** Primer sequences for real-time quantitative polymerase chain reaction.

Primer	Sequence	Product size	GenBank accession number
*PPAR*γ^1^	5′-CCA CTG CAG GAA CAG AAC AA-3′ (forward)	249 bp^7^	XM_010718432.1
	5′-CTC CCG TGT CAT GAA TCC TT-3′ (reverse)		
*C/EBP*β^2^	5′-GCA CAG CGA CGA GTA CAA G-3′ (forward)	82 bp	XM_003212165.2
	5′-GTT GCG CAT TTT GGC TTT GTC-3′ (reverse)		
*NPY*^3^	5′-CCC AGA GAC ACT GAT CTC AGA C-3′ (forward)	76 bp	XM_010712774.3
	5′-AGG GTC TTC AAA CCG GGA TCT-3′ (reverse)		
*KLF1*^4^	5′-CCC CGA CAT GAT GCA CAG GAT-3′ (forward)	157 bp	XM_422416.6
	5′-AGG CAG AGG GTA ATT GGG GC-3′ (reverse)		
*KLF7*^5^	5′-CCA TTG TGA CAG GTG TTT TTC C-3′ (forward)	152 bp	XM_010713480.2
	5′-TCT TGC AAG GCA GCA CAT TAT-3′ (reverse)		
*GAPDH*^6^	5′-GAG GGT AGT GAA GGC TGC TG-3′ (forward)	200 bp	U94327.1
	5′-CCA CAA CAC GGT TGC TGT AT-3′ (reverse)		

*^1^*PPAR*γ, Peroxisome proliferator-activated receptor gamma.*

*^2^*C/EBP*β, CCAAT/enhancer-binding protein beta.*

*^3^*NPY*, Neuropeptide Y.*

*^4^*KLF1*, Kruppel Like Factor 1.*

*^5^*KLF7*, Kruppel Like Factor 7.*

*^6^*GAPDH*, Glyceraldehyde-3-phosphate dehydrogenase.*

*^7^bp, number of base pairs.*

### Statistical Model and Analysis

Data from the AdipoRed assay and RT-qPCR were analyzed as a completely randomized model at each sampling time in SAS (SAS 9.4, SAS Institute Inc., Cary, NC, United States). Main effect of temperature, main effect of cell line, interaction effect between temperature and cell line, and random effect of repeat experiment were included in this model. The statement of Least Square Means in the MIXED procedure was used to determine each mean value and the standard error of the mean (SEM). Differences between each mean were separated with the Pdiff option. Line effect within each treatment group and temperature effect within each cell line was determined with the SLICE option at each sampling time. For the AdipoRed assay, the REG procedure was used to evaluate the linear relationship between sampling times and optical densities of AdipoRed per cell line per treatment group. Difference in the linear relationship was determined with the Contrast statement. *P* ≤ 0.05 was considered as statistically significant.

## Results

### Effect of Thermal Stress and Selection for Growth on Intracellular Lipid Accumulation During Satellite Cell Proliferation

The effect of heat stress (43°C) on intracellular lipid accumulation in pmSCs from the RBC2 and NC turkeys was measured every 24 h during 72 h of proliferation (Table 3). Lipid content in both cell lines increased linearly throughout the duration of proliferation at both 38°C (NC slope: 5.11, RBC2 slope: 2.17) and 43°C (NC slope: 30.50, RBC2 slope: 17.85). The NC line had a greater increase (slope) in lipid content as a function of time at 38°C (*P* < 0.001) and 43°C (*P* = 0.003) than the RBC2 line. At 24 h, the NC line showed a significant increase (*P* < 0.001) in lipid content at 43°C than at 38°C. At 48 and 72 h, lipid content of both the RBC2 (*P* ≤ 0.001) and NC (*P* < 0.001) lines was greater at 43°C than at 38°C. Lipid content of the RBC2 and NC lines increased 6.35-fold (*P* < 0.001) and 4.73-fold (*P* < 0.001) at 43°C than at 38°C at 72 h of proliferation, respectively. A significant interaction effect between temperature and cell line was observed at 24 h (*P* = 0.002) and 72 h (*P* = 0.041).

**TABLE 3:** Effect of heat stress (43°C) on lipid accumulation during proliferation of satellite cells isolated from the pectoralis major muscle of Randombred Control Line 2 (RBC2) and Nicholas Commercial (NC) turkeys^1^.

Line	Temperature (°C)	Sampling time				*P*-value^2^
		0 h	24 h	48 h	72 h	
RBC2	38	0.06^a,x^ ± 0.00	0.08^c,yx^ ± 0.00	0.09^c,y^ ± 0.02	0.23^c,z^ ± 0.10	<0.001
	43	0.06^a,y^ ± 0.00	0.08^bc,y^ ± 0.00	0.17^b,y^ ± 0.02	1.46^b,z^ ± 0.10	<0.001
NC	38	0.06^a,y^ ± 0.00	0.12^a,y^ ± 0.00	0.12^bc,y^ ± 0.02	0.44^c,z^ ± 0.10	<0.001
	43	0.06^a,y^ ± 0.00	0.10^b,y^ ± 0.00	0.24^a,y^ ± 0.02	2.08^a,z^ ± 0.10	<0.001

*P*-values^3^	L	0.552	<0.001	0.002	<0.001	
	T	0.070	0.082	<0.001	<0.001	
	L × T	0.351	0.002	0.211	0.041	

*^1^Value represents mean of AdipoRed optical density (OD/well) at 485 nm ± Standard error of mean (SEM).*

*^2^Effect of sampling time within each temperature and cell line.*

*^3^Effect of line (L), temperature (T), and line by temperature interaction (L × T) within each sampling time.*

*^a–c^Mean of AdipoRed OD (mean ± SEM) within a column (sampling time) without a common letter are significantly different.*

*^x–z^Mean of AdipoRed OD (mean ± SEM) within a row (temperature and cell line) without a common letter are significantly different.*

**P* ≤ 0.05 was considered as significantly different.*

The effect of cold stress (33°C) on intracellular lipid accumulation in pmSCs from both lines was measured every 24 h during 72 h of proliferation (Table 4). Lipid content in pmSCs from both lines was slightly higher (*P* < 0.001) at 24 and 72 h than at 0 h at 33°C. At 48 and 72 h, both lines had lower lipid content (*P* < 0.001) at 33°C than at 38°C. Lipid content in the RBC2 and NC lines decreased 4.00-fold (*P* = 0.003) and 11.83-fold (*P* < 0.001) at 33°C compared to 38°C at 72 h of proliferation. There was a significant interaction effect between temperature and cell line at 24 h (*P* = 0.014), 48 h (*P* = 0.004), and 72 h (*P* < 0.001).

**TABLE 4:** Effect of cold stress (33°C) on lipid accumulation during proliferation of satellite cells isolated from the pectoralis major muscle of Randombred Control Line 2 (RBC2) and Nicholas Commercial (NC) turkeys^1^.

Line	Temperature (°C)	Sampling time				*P*-value^2^
		0 h	24 h	48 h	72 h	
RBC2	38	0.05^a,x^ ± 0.00	0.05^by,x^ ± 0.00	0.08^b,y^ ± 0.01	0.24^b,z^ ± 0.04	<0.001
	33	0.04^ab,y^ ± 0.00	0.07^a,z^ ± 0.00	0.05^c,y^ ± 0.01	0.06^c,zy^ ± 0.04	<0.001
NC	38	0.04^b,y^ ± 0.00	0.06^a,y^ ± 0.00	0.10^a,y^ ± 0.01	0.71^a,z^ ± 0.04	<0.001
	33	0.05^ab,zy^ ± 0.00	0.06^ab,z^ ± 0.00	0.03^c,y^ ± 0.01	0.06^c,z^ ± 0.04	0.009

*P*-values^3^	L	0.136	0.695	0.726	<0.001	
	T	0.822	0.190	<0.001	<0.001	
	L × T	0.110	0.014	0.004	<0.001	

*^1^Value represents mean of AdipoRed optical density (OD/well) at 485 nm ± Standard error of mean (SEM).*

*^2^Effect of sampling time within each temperature and cell line.*

*^3^Effect of line (L), temperature (T), and line by temperature interaction (L × T) within each sampling time.*

*^a–c^Mean of AdipoRed OD (mean ± SEM) within a column (sampling time) without a common letter are significantly different.*

*^x–z^Mean of AdipoRed OD (mean ± SEM) within a row (temperature and cell line) without a common letter are significantly different.*

**P* ≤ 0.05 was considered as significantly different.*

### Effect of Thermal Stress and Selection for Growth on Intracellular Lipid Accumulation During Satellite Cell Differentiation

A heat stress (43°C) was administrated during the 72 h of proliferation and/or during the 72 h of differentiation. Treatment groups were 38°→43°C, 43°→38°C, and 43°→43°C, and these groups were compared to the 38°→38°C control group. Lipid content in pmSCs from both the RBC2 and NC lines was measured at 24, 48, and 72 h of differentiation (Table 5). At 24 h, a lower amount of lipid content was measured only in the NC line in the 38°→43°C group (1.52-fold, *P* = 0.007) than the 38°→38°C group, and no significant change in lipid content occurred in the RBC2 line (*P* = 0.329). Lipid content in the RBC2 and NC lines was 2.21-fold (*P* < 0.001) and 2.36-fold (*P* < 0.001) higher in the 43°→38°C group than the 38°→38°C group at 24 h. In the 43°→43°C group at 24 h, lipid content was 1.60-fold (*P* = 0.002) and 1.42-fold (*P* < 0.001) higher in the RBC2 and NC lines than the 38°→38°C group. Line effect was significant in the 38°→38°C (*P* = 0.005), 43°→38°C (*P* < 0.001), and 43°→43°C (*P* = 0.002) groups at 24 h. A significant interaction effect between temperature and cell line was observed at 24 h (*P* < 0.001). At 48 h, no significant difference in lipid content was observed in either line (*P* > 0.118) between the 38°→43°C and 38°→38°C groups. Lipid content was greater in the 43°→38°C group in both lines (*P* < 0.001) than the 38°→38°C group at 48 h. Greater lipid content was observed only in the NC line (*P* = 0.002) in the 43°→43°C group than the 38°→38°C group at 48 h. A significant line effect was observed in the 43°→38°C (*P* < 0.001) and 43°→43°C (*P* = 0.017) groups at 48 h. An interaction effect between temperature and cell line was not significant at 48 h (*P* = 0.179). At 72 h, no significant difference in lipid content was observed in either line in 38°→43°C (*P* > 0.643) and 43°→43°C (*P* > 0.154) groups than the 38°→38°C group. A slightly greater amount of lipid (1.53-fold, *P* = 0.003) was observed only in the RBC2 line in the 43°→38°C group than the 38°→38°C group at 72 h. Line effect was significant in the 38°→38°C (*P* = 0.005) and 38°→43°C (*P* = 0.003) groups at 72 h. No significant interaction effect was observed between temperature and cell line at 72 h (*P* = 0.698). From 24 to 72 h, a linear decrease in lipid content was observed in both lines in the 38°→38°C, 43°→38°C and 43°→43°C groups. In the 38°→38°C group, the NC line had a steeper linear reduction in lipid content than the RBC2 line (NC slope: −9.05, RBC2 slope: −5.56, *P* < 0.001). Lipid content in the NC line showed greater reduction than the RBC2 line in the 43°→38°C group (NC slope: −26.99, RBC2, slope: −14.35, *P* < 0.001). In the 43°→43°C group, both lines had a linear decrease in lipid content from 24 to 72 h (NC slope: −16.89, RBC2 slope: −11.79), and there was no line difference (*P* = 0.067).

**TABLE 5:** Effect of heat stress (43°C) on lipid accumulation during differentiation of satellite cells isolated from the pectoralis major muscle of Randombred Control Line 2 (RBC2) and Nicholas Commercial (NC) line turkeys^1^.

Line	Treatment group^2^	Sampling time			*P*-value^3^
		24 h	48 h	72 h	
RBC2	38 → 38	0.43^d,z^ ± 0.06	0.26^def,y^ ± 0.03	0.17^c,y^ ± 0.02	<0.001
	38 → 43	0.35^d,z^ ± 0.06	0.21^f,y^ ± 0.03	0.18^c,y^ ± 0.02	<0.001
	43 → 38	0.95^b,z^ ± 0.06	0.57^b,y^ ± 0.03	0.26^ab,x^ ± 0.02	<0.001
	43 → 43	0.69^c,z^ ± 0.06	0.33^d,y^ ± 0.03	0.15^c,y^ ± 0.03	<0.001
NC	38 → 38	0.67^c,z^ ± 0.06	0.30^de,y^ ± 0.03	0.26^ab,y^ ± 0.02	<0.001
	38 → 43	0.44^d,z^ ± 0.06	0.24^ef,y^ ± 0.03	0.27^ab,y^ ± 0.02	<0.001
	43 → 38	1.58^a,z^ ± 0.06	0.71^a,y^ ± 0.03	0.32^a,x^ ± 0.02	<0.001
	43 → 43	0.95^b,z^ ± 0.06	0.42^c,y^ ± 0.03	0.20^bc,x^ ± 0.03	<0.001

*P*-values^4^	L	<0.001	<0.001	<0.001	
	T	<0.001	<0.001	<0.001	
	L × T	<0.001	0.179	0.698	

*^1^Value represents mean of AdipoRed optical density (OD/well) at 485 nm ± Standard error of mean (SEM).*

*^2^Group name represents incubation temperature (°C) during proliferation → incubation temperature (°C) during differentiation.*

*^3^Effect of sampling time within each temperature and cell line.*

*^4^Effect of line (L), temperature (T), and line by temperature interaction (L × T) within each sampling time.*

*^a–f^Mean of AdipoRed OD (mean ± SEM) within a column (sampling time) without a common letter are significantly different.*

*^x–z^Mean of AdipoRed OD (mean ± SEM) within a row (temperature and cell line) without a common letter are significantly different.*

**P* ≤ 0.05 was considered as significantly different.*

A cold stress (33°C) was administrated during the 72 h of proliferation and/or during the 72 h of differentiation. Treatment groups were 38°→33°C, 33°→38°C, and 33°→33°C, and these groups were compared to the 38°→38°C control group. Lipid content in pmSCs from both lines was measured at 24, 48, and 72 h of differentiation (Table 6). At 24 h, lipid content was lower only in the RBC2 line in the 38°→33°C group (1.27-fold, *P* = 0.035) than the 38°→38°C group. Lipid content in the RBC2 and NC lines was 2.75-fold (*P* < 0.001) and 5.44-fold (*P* < 0.001) lower in the 33°→38°C group than the 38°→38°C group at 24 h. In the 33°→33°C group, lipid content was 2.80-fold (*P* = 0.002) and 6.13-fold (*P* < 0.001) lower in RBC2 and NC lines than the 38°→38°C group at 24 h. Line effects were significant in the 38°→38°C (*P* < 0.001) and 38°→33°C (*P* < 0.001) groups at 24 h. An interaction effect between temperature and cell line was significant at 24 h (*P* < 0.001). At 48 h, a greater amount of lipid was measured only in the NC line (1.45-fold, *P* < 0.001) in the 38°→33°C group than the 38°→38°C group. Lipid content in the RBC2 and NC lines was 2.36-fold (*P* < 0.001) and 2.95-fold (*P* < 0.001) lower in the 33°→38°C group than the 38°→38°C group at 48 h. In the 33°→33°C group, lipid content was 2.51-fold (*P* = 0.002) and 3.20-fold (*P* < 0.001) lower in RBC2 and NC lines than the 38°→38°C group at 48 h. A significant line effect occurred in the 38°→38°C (*P* < 0.001) and 38°→33°C (*P* < 0.001) groups at 48 h. There was an interaction effect between temperature and cell line at 48 h (*P* < 0.001). At 72 h, a slightly greater amount of lipid content was observed only in the RBC2 line in the 38°→33°C group (1.47-fold, *P* < 0.001) than the 38°→38°C group. Lipid content in the RBC2 and NC lines was 2.66-fold (*P* < 0.001) and 3.00-fold (*P* < 0.001) lower in the 33°→38°C group than the 38°→38°C group at 72 h. In the 33°→33°C group, lipid content was 3.48-fold (*P* = 0.002) and 3.56-fold (*P* < 0.001) lower in RBC2 and NC lines than the 38°→38°C group at 72 h. A line effect was significant in the 38°→38°C (*P* = 0.001) and 38°→33°C (*P* < 0.001) groups at 72 h. There was an interaction between the effect of temperature and cell line at 72 h (*P* < 0.001). From 24 to 72 h, the amount of lipid content in both lines was very low throughout the duration of the experiment in the 38°→33°C, 33°→38°C, and 33°→33°C groups. A minor linear reduction in lipid content was only observed in the NC line (slope: −5.06) in the 38°→33°C group from 24 to 72 h.

**TABLE 6:** Effect of cold stress (33°C) on lipid accumulation during differentiation of satellite cells isolated from the pectoralis major muscle of Randombred Control Line 2 (RBC2) and Nicholas Commercial (NC) line turkeys^1^.

Line	Treatment group^2^	Sampling time			*P*-value^3^
		24 h	48 h	72 h	
RBC2	38 → 38	0.31^b,z^ ± 0.02	0.27^c,z^ ± 0.02	0.30^c,z^ ± 0.02	<0.001
	38 → 33	0.24^c,z^ ± 0.02	0.23^c,z^ ± 0.02	0.25^c,z^ ± 0.02	0.649
	33 → 38	0.11^d,z^ ± 0.02	0.12^d,z^ ± 0.02	0.11^d,z^ ± 0.02	0.717
	33 → 33	0.11^d,z^ ± 0.02	0.11^d,z^ ± 0.02	0.09^d,y^ ± 0.02	<0.001
NC	38 → 38	0.79^a,z^ ± 0.02	0.43^b,y^ ± 0.02	0.39^b,y^ ± 0.02	0.007
	38 → 33	0.78^a,z^ ± 0.02	0.62^a,y^ ± 0.02	0.58^a,y^ ± 0.02	<0.001
	33 → 38	0.15^d,z^ ± 0.02	0.14^d,z^ ± 0.02	0.13^d,z^ ± 0.02	0.759
	33 → 33	0.13^d,z^ ± 0.02	0.13^d,z^ ± 0.02	0.11^d,y^ ± 0.02	0.023

*P*-values^4^	L	<0.001	<0.001	<0.001	
	T	<0.001	<0.001	<0.001	
	L × T	<0.001	<0.001	<0.001	

*^1^Value represents mean of AdipoRed optical density (OD/well) at 485 nm ± Standard error of mean (SEM).*

*^2^Group name represents incubation temperature (°C) during proliferation → incubation temperature (°C) during differentiation.*

*^3^Effect of sampling time within each temperature and cell line.*

*^4^Effect of line (L), temperature (T), and line by temperature interaction (L × T) within each sampling time.*

*^a–d^Mean of AdipoRed OD (mean ± SEM) within a column (sampling time) without a common letter are significantly different.*

*^y–z^Mean of AdipoRed OD (mean ± SEM) within a row (temperature and cell line) without a common letter are significantly different.*

**P* ≤ 0.05 was considered as significantly different.*

### Effect of Thermal Stress and Selection for Growth on Expression of Adipogenic Regulatory Genes

**FIGURE 1:**
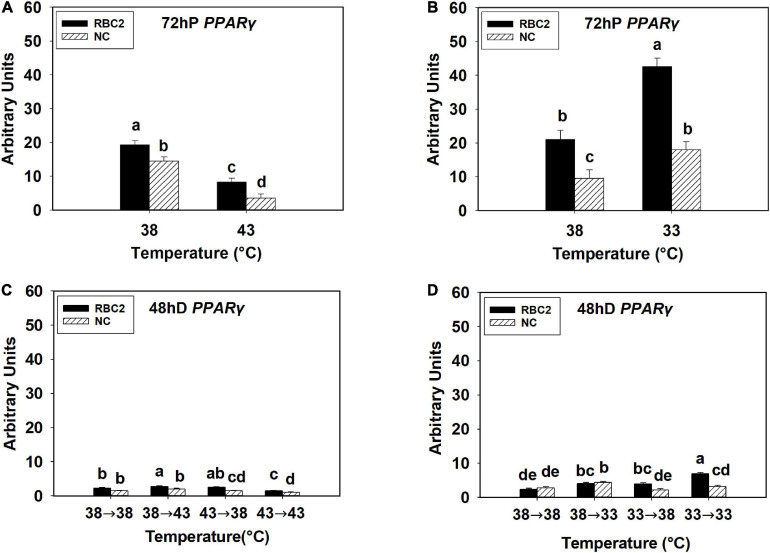
Expression of peroxisome proliferator-activated receptor gamma (*PPAR*γ) at 72 h of proliferation (72 hP) and 48 h of differentiation (48 hD) in satellite cells isolated from the pectoralis major (p. major) muscle of 7-day-old Randombred Control Line 2 (RBC2) and 7-day-old Nicholas Commercial (NC) turkeys. **(A)** Satellite cell proliferation at 38° or 43°C for 72 h. **(B)** Satellite cell proliferation at 38° or 33°C for 72 h. **(C)** Satellite cell proliferation for 72 h and differentiation for 48 h, both at 38°C (38°→38°C); proliferation at 38°C for 72 h and differentiation at 43°C for 48 h (38°→43°C); proliferation at 43°C for 72 h and differentiation at 38°C for 48 h (43°→38°C); or proliferation for 72 h and differentiation for 48 h, both at 43°C (43°→43°C). **(D)** Satellite cell proliferation for 72 h and differentiation for 48 h, both at 38°C (38°→38°C); proliferation at 38°C for 72 h and differentiation at 33°C for 48 h (38°→33°C); proliferation at 33°C for 72 h and differentiation at 38°C for 48 h (33°→38°C); or proliferation for 72 h and differentiation for 48 h, both at 33°C (33°→33°C). Each graph bar represents a mean arbitrary unit, and each error bar represents a standard error of the mean. Mean values without a same letter were significantly different (*P* ≤ 0.05).

At 72 h of proliferation, *PPAR*γ expression was lower (*P* < 0.010) in the NC line than the RBC2 line at 38°C (Figures 1A,B). The expression of *PPAR*γ at 43°C was decreased 4.06-fold (*P* < 0.001) and 2.32-fold (*P* < 0.001) in the NC and RBC2 lines, respectively, compared to 38°C at 72 h of proliferation (Figure 1A). With cold stress (33°C), the expression of *PPAR*γ was increased 2.02-fold (*P* < 0.001) and 1.88-fold (*P* = 0.021) in both the RBC2 and NC lines compared to the 38°C at 72 h of proliferation (Figure 1B). A significant interaction effect between temperature and cell line was observed at 33°C (*P* = 0.015, Figure 1B). At 48 h of differentiation, there was no difference in *PPAR*γ expression between the two cell lines in the 38°→38°C group (Figures 1C,D). The expression of *PPAR*γ was higher only in the RBC2 line (1.22-fold, *P* = 0.012) in the 38°→43°C group than the 38°→38°C group (Figure 1C). In the 43°→38°C group, *PPAR*γ expression was reduced only in the NC line than the 38°→38°C group (1.41-fold, *P* < 0.001, Figure 1C). Both the RBC2 (1.53-fold, *P* < 0.001) and NC (1.86-fold, *P* < 0.001) lines showed a lower expression of *PPAR*γ in the 43°→43°C group than the 38°→38°C group (Figure 1C). With the cold stress, *PPAR*γ expression was upregulated in both the RBC2 (1.69-fold, *P* = 0.002) and NC (1.57-fold, *P* = 0.003) lines in the 38°→33°C group than the 38°→38°C group (Figure 1D). Higher expression of *PPAR*γ occurred only in the RBC2 line SCs in the 33°→38°C (1.66-fold, *P* = 0.002) and 33°→33°C (2.91-fold, *P* < 0.001) groups than the 38°→38°C group at 48 h of differentiation (Figure 1D). Interaction effect between temperature and cell line was significant among the cold treatment groups (*P* < 0.001, Figure 1D).

**FIGURE 2:**
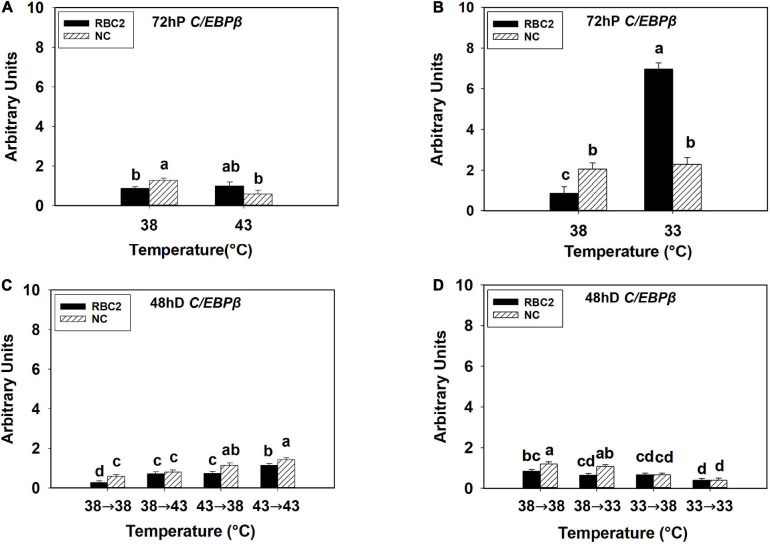
Expression of CCAAT/enhancer-binding protein beta (*C/EBP*β) at 72 h of proliferation (72 hP) and 48 h of differentiation (48 hD) in satellite cells isolated from the pectoralis major (p. major) muscle of 7-day-old Randombred Control Line 2 (RBC2) and 7-day-old Nicholas Commercial (NC) turkeys. **(A)** Satellite cell proliferation at 38° or 43°C for 72 h. **(B)** Satellite cell proliferation at 38° or 33°C for 72 h. **(C)** Satellite cell proliferation for 72 h and differentiation for 48 h, both at 38°C (38°→38°C); proliferation at 38°C for 72 h and differentiation at 43°C for 48 h (38°→43°C); proliferation at 43°C for 72 h and differentiation at 38°C for 48 h (43°→38°C); or proliferation for 72 h and differentiation for 48 h, both at 43°C (43°→43°C). **(D)** Satellite cell proliferation for 72 h and differentiation for 48 h, both at 38°C (38°→38°C); proliferation at 38°C for 72 h and differentiation at 33°C for 48 h (38°→33°C); proliferation at 33°C for 72 h and differentiation at 38°C for 48 h (33°→38°C); or proliferation for 72 h and differentiation for 48 h, both at 33°C (33°→33°C). Each graph bar represents a mean arbitrary unit, and each error bar represents a standard error of the mean. Mean values without a same letter were significantly different (*P* ≤ 0.05).

At 72 h of proliferation, *C/EBP*β was upregulated (*P* < 0.001) in the NC line than the RBC2 line at 38°C (Figures 2A,B). The expression of *C/EBP*β was reduced 2.23-fold in the NC line (*P* = 0.003) whereas no difference (*P* = 0.593) was observed in the RBC2 line at 43°C compared to the 38°C at 72 h of proliferation (Figure 2A). With the cold stress of 33°C, a dramatic increase in *C/EBP*β expression was observed in the RBC2 line (8.12-fold, *P* < 0.001) compared to the 38°C treatment at 72 h of proliferation, and no significant change (*P* = 0.603) was observed in the NC line (Figure 2B). There was a significant interaction effect between temperature and cell line at both 43°C (*P* = 0.006) and 33°C (*P* < 0.001, Figures 2A,B). At 48 h of differentiation, the expression of *C/EBP*β was higher in the NC line (*P* = 0.007) than the RBC2 line in the 38°→38°C group (Figures 2C,D). In the 38°→43°C group, *C/EBP*β expression was upregulated only in the RBC2 line (2.62-fold, *P* < 0.001) than the 38°→38°C group (Figure 2C). Both the RBC2 and NC lines showed higher *C/EBP*β expression in the 43°→38°C (NC: 1.94-fold, *P* < 0.001; RBC2: 2.68-fold, *P* < 0.001) and 43°→43°C (NC: 2.43-fold, *P* < 0.001, RBC2: 4.16-fold, *P* < 0.001) groups than the 38°→38°C group at 48 h of differentiation (Figure 2C). With the cold stress, the NC line showed a lower *C/EBP*β expression in both the 33°→38°C (1.84-fold, *P* < 0.001) and 33°→33°C (3.06-fold, *P* < 0.001) groups than the 38°→38°C group (Figure 2D). In the RBC2 line, the expression of *C/EBP*β was lower only in the 33°→33°C group (2.15-fold, *P* = 0.002) than the 38°→38°C group (Figure 2D). Interaction effect between temperature and cell line was significant among the cold treatment groups (*P* = 0.042, Figure 2D).

**FIGURE 3:**
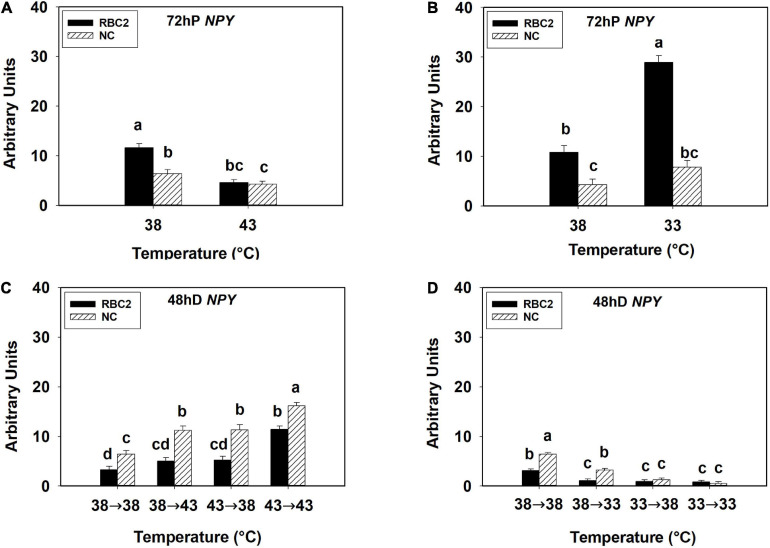
Expression of neuropeptide Y (*NPY*) at 72 h of proliferation (72 hP) and 48 h of differentiation (48 hD) in satellite cells isolated from the pectoralis major (p. major) muscle of 7-day-old Randombred Control Line 2 (RBC2) and 7-day-old Nicholas Commercial (NC) turkeys. **(A)** Satellite cell proliferation at 38° or 43°C for 72 h. **(B)** Satellite cell proliferation at 38° or 33°C for 72 h. **(C)** Satellite cell proliferation for 72 h and differentiation for 48 h, both at 38°C (38°→38°C); proliferation at 38°C for 72 h and differentiation at 43°C for 48 h (38°→43°C); proliferation at 43°C for 72 h and differentiation at 38°C for 48 h (43°→38°C); or proliferation for 72 h and differentiation for 48 h, both at 43°C (43°→43°C). **(D)** Satellite cell proliferation for 72 h and differentiation for 48 h, both at 38°C (38°→38°C); proliferation at 38°C for 72 h and differentiation at 33°C for 48 h (38°→33°C); proliferation at 33°C for 72 h and differentiation at 38°C for 48 h (33°→38°C); or proliferation for 72 h and differentiation for 48 h, both at 33°C (33°→33°C). Each graph bar represents a mean arbitrary unit, and each error bar represents a standard error of the mean. Mean values without a same letter were significantly different (*P* ≤ 0.05).

At 72 h of proliferation, *NPY* expression was lower (*P* < 0.005) in the NC line than the RBC2 line at 38°C (Figures 3A,B). The expression of *NPY* was downregulated in both the RBC2 (2.53-fold, *P* < 0.001) and NC (1.49-fold, *P* = 0.049) lines at 43°C compared to the 38°C at 72 h of proliferation (Figure 3A). At 33°C, the expression of *NPY* was increased 2.67-fold (*P* < 0.001) in the RBC2 line while no significant change (*P* = 0.056) was observed in the NC line compared to the 38°C at 72 h of proliferation (Figure 3B). A significant interaction effect between temperature and cell line was observed at both 43°C (*P* = 0.003) and 33°C (*P* < 0.001, Figures 3A,B). At 48 h of differentiation, *NPY* expression was higher in the NC line (*P* = 0.002) than the RBC2 line in the 38°→38°C group (Figures 3C,D). The expression of *NPY* was higher in the NC line in the 38°→43°C (1.74-fold, *P* < 0.001), 43°→38°C (1.76-fold, *P* < 0.001), and 43°→43°C (2.50-fold, *P* < 0.001) groups than the 38°→38°C group (Figure 3C). Greater *NPY* expression was observed in the RBC2 line (3.47-fold, *P* < 0.001) only in the 43°→43°C group than the 38°→38°C group (Figure 3C). With the cold stress, *NPY* expression was lower in both the RBC2 (*P* < 0.001) and NC (*P* < 0.001) lines in the 38°→33°C (NC: 3.98-fold, RBC2: 2.77-fold), 33°→38°C (NC: 10.48-fold, RBC2: 3.43-fold), and 33°→33°C (NC: 24.41-fold, RBC2: 3.69-fold) groups than the 38°→38°C group (Figure 3D). Interaction effect between temperature and cell line was significant among the cold treatment groups (*P* < 0.001, Figure 3D).

**FIGURE 4:**
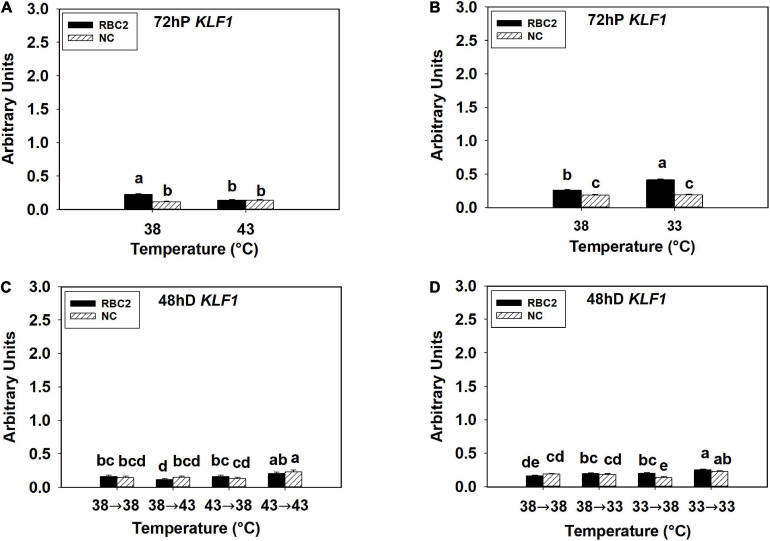
Expression of kruppel like factor 1 (*KLF1*) at 72 h of proliferation (72 hP) and 48 h of differentiation (48 hD) in satellite cells isolated from the pectoralis major (p. major) muscle of 7-day-old Randombred Control Line 2 (RBC2) and 7-day-old Nicholas Commercial (NC) turkeys. **(A)** Satellite cell proliferation at 38° or 43°C for 72 h. **(B)** Satellite cell proliferation at 38° or 33°C for 72 h. **(C)** Satellite cell proliferation for 72 h and differentiation for 48 h, both at 38°C (38°→38°C); proliferation at 38°C for 72 h and differentiation at 43°C for 48 h (38°→43°C); proliferation at 43°C for 72 h and differentiation at 38°C for 48 h (43°→38°C); or proliferation for 72 h and differentiation for 48 h, both at 43°C (43°→43°C). **(D)** Satellite cell proliferation for 72 h and differentiation for 48 h, both at 38°C (38°→38°C); proliferation at 38°C for 72 h and differentiation at 33°C for 48 h (38°→33°C); proliferation at 33°C for 72 h and differentiation at 38°C for 48 h (33°→38°C); or proliferation for 72 h and differentiation for 48 h, both at 33°C (33°→33°C). Each graph bar represents a mean arbitrary unit, and each error bar represents a standard error of the mean. Mean values without a same letter were significantly different (*P* ≤ 0.05).

The *KLF1* expression was low in all the hot and cold treatment groups in both lines at both 72 h of proliferation and 48 h of differentiation (Figures 4A–D). At 72 h of proliferation, *KLF1* had greater expression in the RBC2 line (*P* < 0.001) than the NC line at 38°C (Figures 4A,B). The expression of *KLF1* was lower only in the RBC2 (1.66-fold, *P* < 0.001) line at 43°C compared to 38°C (Figure 4A). There was a significant interaction effect between temperature and cell line at 43°C (*P* < 0.001, Figure 4A). At the cold temperature of 33°C, *KLF1* expression was higher in the RBC2 line (1.59-fold, *P* < 0.001) while no significant (*P* = 0.659) difference was observed in the NC line compared to the 38°C at 72 h of proliferation (Figure 4B). At 48 h of differentiation, no difference in *KLF1* expression between the two cell lines (*P* = 0.473) was observed in the 38°→38°C group (Figures 4C,D). In the 38°→43°C group, reduction in *KLF1* expression occurred only in the RBC2 line (1.44-fold, *P* = 0.045) than the 38°→38°C group (Figure 4C). The expression of *KLF1* expression was slightly higher in both lines (NC: 1.59-fold, *P* < 0.01; RBC2: 1.23-fold, *P* < 0.01) in the 43°→43°C group than the 38°→38°C group (Figure 4C). With the cold stress, *KLF1* expression was upregulated only in the RBC2 line in the 38°→33°C (1.23-fold, *P* = 0.033) and 33°→38°C (1.24-fold, *P* = 0.023) groups than the 38°→33°C group (Figure 4D). In the 33°→33°C group, both the RBC2 (1.59-fold, *P* < 0.001) and NC (1.22-fold, *P* = 0.017) lines had higher expression of *KLF1* than the 38°→33°C group (Figure 4D). There was a significant interaction effect between temperature and cell line among the cold treatment groups (*P* < 0.001, Figure 4D).

**FIGURE 5:**
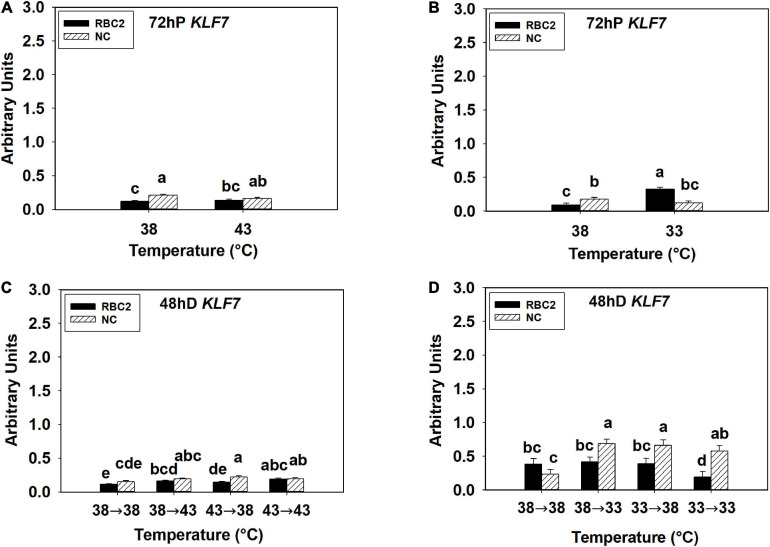
Expression of kruppel like factor 7 (*KLF7*) at 72 h of proliferation (72 hP) and 48 h of differentiation (48 hD) in satellite cells isolated from the pectoralis major (p. major) muscle of 7-day-old Randombred Control Line 2 (RBC2) and 7-day-old Nicholas Commercial (NC) turkeys. **(A)** Satellite cell proliferation at 38° or 43°C for 72 h. **(B)** Satellite cell proliferation at 38° or 33°C for 72 h. **(C)** Satellite cell proliferation for 72 h and differentiation for 48 h, both at 38°C (38°→38°C); proliferation at 38°C for 72 h and differentiation at 43°C for 48 h (38°→43°C); proliferation at 43°C for 72 h and differentiation at 38°C for 48 h (43°→38°C); or proliferation for 72 h and differentiation for 48 h, both at 43°C (43°→43°C). **(D)** Satellite cell proliferation for 72 h and differentiation for 48 h, both at 38°C (38°→38°C); proliferation at 38°C for 72 h and differentiation at 33°C for 48 h (38°→33°C); proliferation at 33°C for 72 h and differentiation at 38°C for 48 h (33°→38°C); or proliferation for 72 h and differentiation for 48 h, both at 33°C (33°→33°C). Each graph bar represents a mean arbitrary unit, and each error bar represents a standard error of the mean. Mean values without a same letter were significantly different (*P* ≤ 0.05).

The overall expression of *KLF7* was low in all the hot and cold treatment groups in both lines at both 72 h of proliferation and 48 h of differentiation (Figures 5A–D). At 72 h of proliferation, the NC line had higher expression of *KLF7* (*P* < 0.001) than the RBC2 line at 38°C (Figures 5A,B). The heat stress had no significant effect on *KLF7* expression (*P* = 0.434) in SCs from both lines at 72 h of proliferation (Figure 5A). The cold stress upregulated *KLF7* expression in the RBC2 line (3.5-fold, *P* < 0.001) but not in the NC line (*P* = 0.175) at 72 h of proliferation (Figure 5B). A significant interaction effect between temperature and cell line was observed at 33°C (*P* < 0.001, Figure 5B). At 48 h of differentiation, no significant difference in *KLF7* expression (*P* = 0.087) was observed between the two cell lines in the 38°→38°C group (Figures 5C,D). The expression of *KLF7* was higher only in the RBC2 line in the 38°→43°C (1.44-fold, *P* = 0.004) and 43°→43°C (1.64-fold, *P* < 0.001) groups than the 38°→38°C group (Figure 5C). The expression of *KLF7* slightly upregulated in both the RBC2 (1.32-fold, *P* = 0.023) and the NC (1.48-fold, *P* = 0.004) lines in the 43°→38°C group than the 38°→38°C group (Figure 5C). With the cold treatment, higher *KLF7* expression occurred only in the NC line in the 38°→33°C (2.92-fold, *P* < 0.001) and 33°→38°C (2.81-fold, *P* < 0.001) groups than the 38°→38°C group (Figure 5D). In the 33°→33°C group, *KLF7* expression increased in the NC line (2.45-fold, *P* = 0.004) but decreased in the RBC2 line (2.51-fold, *P* = 0.015) than the 38°→38°C group (Figure 5D). Interaction effect between temperature and cell line was significant among the cold treatment groups (*P* = 0.001, Figure 5D).

## Discussion

Myogenic SCs are partially differentiated stem cells with multiple cellular fates (Asakura et al., 2001; Shefer et al., 2004) that respond to extrinsic stimuli including oxygen concentration (Csete et al., 2001; Redshaw and Loughna, 2012), nutritional status (Powell et al., 2014), and environmental temperature (Harding et al., 2015; Clark et al., 2017) resulting in changes in SC adipogenic characteristics (adipogenic gene expression or lipid synthesis). Post-hatch heat and cold stress has been shown to affect intramuscular fat content in the poultry breast muscle (Zhang et al., 2012; Piestun et al., 2017; Patael et al., 2019). The changes in intramuscular fat deposition may be, in part, attributable to the altered adipogenic potential of SCs (Smith and Johnson, 2014; Piestun et al., 2017; Patael et al., 2019).

During the immediate 7 days after hatch, SCs exhibit peak mitotic activity (Mozdziak et al., 1994; Halevy et al., 2000) and responsiveness to extrinsic temperatures (Halevy et al., 2001; Patael et al., 2019; Xu et al., 2021). Furthermore, selection for growth has increased the proliferation (Merly et al., 1998; Velleman et al., 2000) and thermal sensitivity (Clark et al., 2016; Xu et al., 2021) of pmSCs. Rossi et al. (2010) showed that faster-proliferating SCs have a greater potential to spontaneously express adipogenic characteristics than slower-proliferating populations. Thus, the present study determined the extent to which selection for higher growth rate and increased breast muscle yield at a processing age has altered the adipogenic potential of cultured 7-day-old pmSCs, as well as to identify differential adipogenic gene expression in pmSCs following a hot or cold thermal stress. A previous study by Clark et al. (2017) assessed the effect of both temperature and selection for growth on the adipogenic properties of 7-week-old pmSCs from non-commercial F-line turkeys that were selected from the RBC2 line only for increased 16-week body weight. The current study extends the previous work by comparing 7-day-old pmSCs from a contemporary commercial line with that of the RBC2 line. In addition, the effect of timing of the thermal stress during proliferation and/or differentiation on adipogenic potential of pmSCs was also examined.

Due to an asymmetric division strategy, not all daughter cells are committed to a myogenic pathway during proliferation (Kuang et al., 2007). Thus, proliferation is a critical period in which the cellular fate of SCs is determined by environmental temperature. Xu et al. (2021) showed that both hot and cold temperatures had a greater effect on myogenic differentiation if the thermal stress occurred during proliferation rather than only during differentiation. In the present study, heat-treated pmSCs from both lines had more intracellular lipid during late proliferation than late differentiation. In support of this finding, Clark et al. (2017) reported increased lipid synthesis in 7-week-old turkey pmSCs with hot temperatures during proliferation. Lipid content of both lines was increased to a higher extent during the earlier stages of differentiation (24 and 48 h) if the heat stress was applied during proliferation than only during differentiation. Heat stress-induced increases in intracellular lipid have been shown to result in a concomitant increase in intramuscular fat content and to have a prolonged effect on the structure and composition of the breast muscle (Piestun et al., 2017; Patael et al., 2019; Halevy, 2020).

Under cold temperatures, intracellular lipid decreased in both lines with the NC line showing a greater reduction than the RBC2 line during proliferation. In previous *in vitro* studies, a reduction in lipid content was observed in pmSCs from both chickens (Harding et al., 2015) and turkeys (Clark et al., 2017) with cold stress. This parallels the results from an *in vivo* study of Patael et al. (2019) that showed no significant intramuscular fat content in the p. major muscle of cold-stressed chickens. The decreased lipid content may arise from reduced pmSC proliferation (Clark et al., 2016; Harding et al., 2016; Xu et al., 2021). Furthermore, if the cold stress was applied during proliferation rather than only during differentiation, the reduction in lipid synthesis was greater in both lines. Thus, lipid synthesis in proliferating pmSCs was more sensitive to both heat and cold stress than differentiating cells. In addition, the lack of detectable expression of the pre-adipocyte markers *KLF1* and *KLF7* during both proliferation and differentiation suggests a lack of pre-adipocytes in the turkey p. major muscle. This is not unexpected as most fat is deposited in abdominal and subcutaneous adipose depots and not as intramuscular fat in poultry (Cahaner et al., 1986; Crespo and Esteve-Garcia, 2002; Fereidoun et al., 2007). This finding also demonstrates the isolated pmSCs used in this study do not contain pre-adipocytes, and lipid synthesis is directly from the SCs. These data are supported by Shefer et al. (2004) who showed the spontaneous conversion of SCs to an adipogenic lineage. Taken together, these results suggest thermal stress may affect the properties of SCs including the synthesis of lipids, and proliferating SCs are more sensitive to temperature stimuli than differentiating SCs. Since poultry skeletal muscle SCs have their highest mitotic activity during the immediate 7 days after hatch (Mozdziak et al., 1994; Halevy et al., 2000), these data support that extrinsic temperature needs to be maintained at an optimal level to avoid possible harmful effects on breast muscle composition.

Another finding in the present study is that selection for growth for increased breast muscling may have enhanced adipogenic differences between the faster-growing NC pmSCs and the slower-growing RBC2 pmSCs. At 38°C during proliferation, the NC line pmSCs had a greater linear increase in lipid content than the RBC2 line. Similar to the findings of Velleman (2014) and Clark et al. (2017), pmSCs from the growth selected F-line turkeys expressed higher levels of the adipogenic genes *PPAR*γ and *C/EBP*α than the RBC2 line during proliferation. The increased adipogenic potential may be partially attributed to the greater proliferation rate of the pmSCs in faster-growing turkeys (Velleman et al., 2000; Clark et al., 2016; Xu et al., 2021). Thus, it is possible that the increased proliferation rates of the growth-selected NC line pmSCs creates a larger SC pool, having a greater potential to express adipogenic properties.

During myogenic differentiation, most SCs are committed to a myogenic pathway, as reflected in the increased myogenic differentiation and enhanced expression of myogenic genes as previously shown (Clark et al., 2016; Xu et al., 2021). This may explain the gradual decrease in lipid synthesis observed in the hot and cold treatment groups in the current study. Expression of *PPAR*γ was significantly reduced during differentiation compared to proliferation in all the treatment groups independent of temperature. However, if the heat stress was continued throughout proliferation and differentiation (43°→43°C), expression of *NPY* and *C/EBP*β increased in both lines compared to other treatment groups (38°→38°C, 38°→43°C, and 43°→38°C), and the NC line showed the highest increase. Taken together with the results by Xu et al. (2021), the NC line pmSCs tended to generate a larger cell pool than the RBC2 line with heat stress. This suggests that a larger pool of SCs may have a greater adipogenic potential even during myogenic differentiation. Furthermore, these results also suggest an intrinsic difference in adipogenic potential between the NC and RBC2 lines during myogenic differentiation, as the NC line pmSCs expressed more *NPY* and *C/EBP*β than the RBC2 line in the control group.

In addition to the intrinsic difference in adipogenic potential, selection for growth may have altered the sensitivity of turkey breast muscle to temperature stimuli. Faster-growing poultry lines tend to have larger diameter myofibers from excessive hypertrophic SC-mediated muscle growth in the breast muscle than slower-growing lines (Wilson et al., 1990; Dransfield and Sosnicki, 1999; Velleman et al., 2003; Joiner et al., 2014). As an anaerobic muscle (Smith and Fletcher, 1988), chicken breast muscle has a reduced capillary supply compared to aerobic muscles, and this is exacerbated in faster-growing chickens (Hoving-Bolink et al., 2000; Yahav, 2000; Havenstein et al., 2003; Joiner et al., 2014). The circulatory supply is necessary to remove lactic acid, the byproduct of anaerobic respiration. The presence of excessively large myofibers typically found in growth-selected breast muscles further limits circulatory supply resulting in breast muscle degradation (Wilson et al., 1990; Velleman et al., 2003). Furthermore, because of a higher metabolic activity (Konarzewski et al., 2000), faster-growing chickens also produce more metabolic heat. Since birds are homeotherms and only keep body temperature within a limited interval (Yahav, 2000, 2015), heat cannot be as readily dissipated due to the reduced circulatory support in faster growing poultry. Therefore, faster-growing poultry has an impaired ability to cope with external thermal stress. In the current study, intracellular lipid synthesis in pmSCs from the faster-growing NC turkeys showed greater variations in response to the heat stress during both proliferation and differentiation than the RBC2 line. However, since the lipid content in pmSCs was higher in the NC line than the RBC2 line at 43°C, the intrinsic difference in lipid content between the two cell lines during proliferation was not affected by the heat stress. In contrast, cold stress eliminated this intrinsic difference between the two cell lines during proliferation. The 7-day-old pmSCs from the NC line tend to have increased myogenic differentiation and wider diameter myotubes than the RBC2 line with heat stress (Clark et al., 2016; Xu et al., 2021). Therefore, if faster-growing turkeys are exposed to a heat stress immediately after hatch, giant muscle fibers coupled with increased intramuscular fat content may occur in the breast muscle, resulting in prolonged negative effects on breast muscle structure and composition.

Adipogenic regulatory genes mediate the response to a thermal stress. The expression of both *NPY* and *PPAR*γ expression was downregulated in the NC line during proliferation than the RBC2 line at the control temperature, and the expression of *NPY* and *PPAR*γ was decreased with heat treatment during proliferation in both lines. A reduction in *PPAR*γ and *C/EBP*β expression in the heat-treated proliferating pmSCs was observed by Clark et al. (2017) as well. In chickens, a high fat diet downregulated *NPY*, *PPAR*γ, and *C/EBP*α expression in both abdominal and subcutaneous adipose tissue (Wang et al., 2017). Furthermore, obesity resistance can be stimulated with a diet that is rich in fat and inhibits the expression of *NPY* and *PPAR*γ in a rodent model (Cifani et al., 2015). In the current study, the increased intracellular lipid resulting from heat stress may act as an intracellular signal inhibiting *NPY* expression to suppress further lipid synthesis. As a key adipogenic transcription factor, *NPY* promotes lipid synthesis through the *PPAR*γ pathway in pre-adipocytes in mice (Tang et al., 2015) and chickens (Zhang et al., 2015; Liu et al., 2017). In turkeys, PPARγ may also be a potential downstream regulator within the NPY-induced adipogenesis pathway in pmSCs, as both *NPY* and *PPAR*γ showed a similar expression pattern at 72 h of proliferation in both lines. Alternatively, with cold stress, the expression of *NPY*, *PPAR*γ, and *C/EBP*β was decreased in the RBC2 line during proliferation. This reduction in expression may be stimulated by low intracellular lipid to prevent further loss of intracellular lipids in the RBC2 line. Furthermore, pmSCs from the NC line appear to have a reduced response to the cold-induced lipid loss than the RBC2 line, as neither *NPY* nor *C/EBP*β showed significant change in the NC line at 33°C compared to the 38°C temperature during proliferation (*PPAR*γ expression only showed a slight increase). Thus, when intracellular lipid was decreased with cold stress, pmSCs from the NC line may have difficulty regulating the lipid loss. This may explain why the cold-treated pmSCs from the NC line had a greater reduction in intracellular lipid than the RBC2 line during proliferation.

In summary, the current study showed that thermal stress alters the adipogenic potential of turkey pmSCs at 7-days of age. During pmSC proliferation, hot temperatures increased the intracellular lipid content in pmSCs while cold temperatures had the opposite effect. Proliferating pmSCs were more sensitive to both heat and cold stress in the determination of intracellular lipid content than differentiating pmSCs. The pmSCs from the faster-growing NC turkeys showed a higher lipid synthesis potential and their cellular fate was more sensitive to both heat and cold stress than the slower-growing RBC2 turkeys. The increased intracellular lipid accumulation of the heat-treated pmSCs may eventually promote intramuscular fat deposition *in vivo*, resulting in prolonged effects on turkey breast muscle structure and composition. Future studies will need to focus on defining the signal transduction pathways involved in the regulation of adipogenesis and their response to heat and cold stress.

## Data Availability Statement

The raw data supporting the conclusions of this article will be made available by the authors, without undue reservation.

## Ethics Statement

Ethical review and approval was not required for the animal study because the study did not use any birds. Cell lines used in the study were previously isolated. Only satellite cells lines were used.

## Author Contributions

All authors have made a substantial and intellectual contribution to the work, and approved the submitted article. SV conceived and designed the experiments with GS and KR. JX performed the experiments and statistical analysis, and wrote the manuscript. SV, GS, and KR revised the manuscript.

## Conflict of Interest

The authors declare that the research was conducted in the absence of any commercial or financial relationships that could be construed as a potential conflict of interest.
